# Dopamine agonist treatment increases sensitivity to gamble outcomes in the hippocampus in *de novo* Parkinson’s disease

**DOI:** 10.1016/j.nicl.2020.102362

**Published:** 2020-07-25

**Authors:** Joyce P.M. van der Vegt, Oliver J. Hulme, Kristoffer H. Madsen, Carsten Buhmann, Bastiaan R. Bloem, Alexander Münchau, Rick C. Helmich, Hartwig R. Siebner

**Affiliations:** a Danish Research Centre for Magnetic Resonance, Copenhagen University Hospital Hvidovre, Hvidovre, Denmark; b Radboud University Medical Centre, Donders Institute for Brain, Cognition and Behaviour, Department of Neurology, Center of Expertise for Parkinson & Movement Disorders, Nijmegen, the Netherlands; c Medisch Spectrum Twente, Department of Neurology, Enschede, the Netherlands; d Department of Applied Mathematics and Computer Science, Technical University of Denmark, Kgs. Lyngby, Denmark; e University Hospital Hamburg-Eppendorf, Department of Neurology, Germany; f Institute of Systems Motor Science, University of Lübeck, Lübeck, Germany; g Copenhagen University Hospital Bispebjerg, Department of Neurology, Copenhagen, Denmark; h Institute for Clinical Medicine, Faculty of Medical and Health Sciences, University of Copenhagen, Copenhagen, Denmark

**Keywords:** fMRI, Reward, Mesolimbic system, Parkinson's disease, Drug-naïve, dopamine-agonist, BDI, beck depression inventory II, BIS, barratt impulsiveness scale, BOLD, blood oxygen level dependent, CI, confidence interval, DA, dopamine, fMRI, functional magnetic resonance imaging, GAQ, gambling addiction questionnaire, GLM, general linear model, ICD, impulse control disorder, MMSE, mini-mental state examination, MNI, Montreal Neurological Institute, PD, Parkinson’s disease, ROI, region of interest, SD, standard deviation, SPM8, statistical parametric mapping 8, SPSS, statistical package for the social sciences, UPDRS, Unified Parkinson's Disease Rating Scale

## Abstract

**Background:**

Parkinson’s disease is associated with severe nigro-striatal dopamine depletion, leading to motor dysfunction and altered reward processing. We previously showed that drug-naïve patients with Parkinson’s disease had a consistent attenuation of reward signalling in the mesolimbic and mesocortical system. Here, we address the neurobiological effects of dopaminergic therapy on reward sensitivity in the mesolimbic circuitry, and how this may contribute to neuropsychiatric symptoms.

**Objectives:**

We tested the hypothesis that (1) dopaminergic treatment would restore the attenuated, mesolimbic and mesocortical responses to reward; and (2) restoration of reward responsivity by dopaminergic treatment would predict motor performance and the emergence of impulse control symptoms.

**Methods:**

In 11 drug-naïve Parkinson patients, we prospectively assessed treatment-induced changes in reward processing before, and eight weeks after initiation of monotherapy with dopamine agonists. They were compared to 10 non-medicated healthy controls who were also measured longitudinally. We used whole-brain functional magnetic resonance imaging at 3 Tesla to assess the reward responsivity of the brain to monetary gains and losses, while participants performed a simple consequential gambling task.

**Results:**

In patients, dopaminergic treatment improved clinical motor symptoms without significantly changing task performance. Dopamine agonist therapy induced a stronger reward responsivity in the right hippocampus with higher doses being less effective. None of the patients developed impulse control disorders in the follow-up period of four years.

**Conclusions:**

Short-term treatment with first-ever dopaminergic medication partially restores deficient reward-related processing in the hippocampus in de novo Parkinson’s disease.

## Highlights

• The effect of first dopaminergic treatment on the reward circuit in PD is unclear.
• Many ON/OFF designs suffer from long-lasting dopaminergic effects (multiple days).
• We tested reward processing in dopa-naive PD before and after treatment initiation.
• Dopaminergic drugs increased reward-related hippocampus activity in PD.
• The relationship with development of neuropsychiatric symptoms remains unknown.

## 1 Introduction

Although Parkinson’s disease (PD) is predominantly categorised as a movement disorder with specific motor symptoms, patients also experience a wide spectrum of non-motor symptoms, which together diminish the patients’ quality of life (Langston, 2006, Gallagher et al., 2010, Soh et al., 2011). Some of these non-motor symptoms have been attributed to adverse effects of dopaminergic treatment (Obeso et al., 2014). Specifically, many patients taking dopamine agonists develop impulse control disorders (ICD), such as hypersexuality, compulsive shopping, punding and pathological gambling (Weintraub et al., 2010). Such aberrant behaviour is thought to be mediated by the maladaptive effect of dopaminergic therapy on the reward system, specifically the midbrain dopaminergic nuclei and their projection sites and several other factors that determine the individual susceptibility (like disease onset and duration, previous mental illness, other addictive behaviour and (epi)genetic factors) (Voon et al., 2017, Grall-Bronnec et al., 2018). According to the “dopamine overdose theory”, treatment with dopamine agonists results in a relative overstimulation of this dopaminergic system, specifically the mesolimbic pathway (Cools et al., 2003). In clinical practice, dopaminergic drug dosage is typically adjusted to maximize the amelioration of classical motoric symptoms caused by nigrostriatal degeneration, but such an approach may inadvertently result in excessive dopaminergic stimulation of the mesolimbic dopaminergic system. According to this theory, the mesolimbic system is vulnerable to this overstimulation because it is relatively spared from the neurodegenerative process early in the disease, and in particular among those patients that are prone to develop ICD in response to dopaminergic treatment (Morrish et al., 1996, Booij et al., 1999, Cools et al., 2003, Braak et al., 2004).

Whilst the theory has appealing explanatory scope, some of its key predictions are yet to be tested. In particular, the neurobiological effects of dopaminergic therapy on reward sensitivity in the mesolimbic circuitry remain to be adequately elucidated. Generally, the effect of dopaminergic treatment on brain function is commonly evaluated by comparing the ON and pragmatic OFF state in patients on long-term dopaminergic medication (Rowe et al., 2008, van Eimeren et al., 2009, Aarts et al., 2014). This approach is problematic because this pragmatic OFF state session is not a true OFF state. This is because dopaminergic medication has a long-term effect that may continue for up to 12 days after treatment cessation (Nutt et al., 1995, Stocchi et al., 2001). Such after-effects are particularly prolonged for dopamine agonists, the class of drugs that is most commonly associated with ICDs. This implies that most ON-OFF state designs are contaminated by the long-term effects of dopaminergic treatment on the reward system. To circumvent this problem, we prospectively assessed treatment-induced changes in reward processing before, and an average of eight weeks after initiation of monotherapy with dopamine agonists and collected data that could influence the individual susceptibility. In 13 drug-naïve PD patients, we used functional magnetic resonance imaging (fMRI) to probe the reward responsivity of the brain to monetary gains and losses, while patients performed a simple consequential gambling task. The task was optimised to entrain high variance in the phasic dopaminergic responses (Mirenowicz and Schultz, 1994). We previously reported the results of the pre-treatment phase in this group of drug-naïve PD patients, where we found a consistent attenuation of reward signalling in the mesolimbic and mesocortical system (van der Vegt et al., 2013). Accordingly, we hypothesised that (1) dopaminergic treatment would restore the attenuated, mesolimbic and mesocortical response to reward. We further hypothesised (2) that the restoration of reward responsivity by dopaminergic treatment would be predictive of motor performance and herald the emergence of impulse control symptoms.

## 2 Materials and Methods

### 2.1 Subjects

Thirteen *de novo* PD patients (8 men, mean age: 58 ± 10 years, median symptom duration ~ 3 years) who were naive to dopaminergic medication (PD_OFF_ condition, henceforth) were recruited (Table 1). In the PD_OFF_ condition, all subjects engaged in a two-outcome monetary gambling task during fMRI data acquisition, reported previously as a standalone experiment (van der Vegt et al., 2013). Immediately after this task, the patients received an acute dopamine challenge and were scanned again whilst performing the same task (these data are not reported in this paper). After this session, all patients started chronic pharmacological treatment with dopamine agonists. Eleven of the thirteen patients (6 men, mean age: 59.2 ± 9.7 years) underwent an additional fMRI experiment after 6–12 weeks of treatment (mean treatment duration: 8.4 ± 2.3 weeks), performing the same gambling task (PD_ON_ condition, henceforth). One patient could not participate in the second session due to knee surgery, and another patient’s data were unsuitable for analyses because of technical problems which caused loss of data files in the transaction from scanner to the data server. Our design was optimized to study the longitudinal effects of chronic dopaminergic treatment, which was optimally dosed for each individual patient during a 6–12 weeks period, and which we compared to both the PD_OFF_ condition and to repeated measurements in healthy controls (Session 1 versus 2).

**Table 1:** Demographics, test scores, and task performance of Parkinson’s patients; unmedicated, on dopamine agonists and at four years follow-up and controls; session 1 and 2.

	Patients				Controls		
	PD_OFF_ T = 0	PD_ON_ T = 6–12 weeks	PD_Follow-up_ T = 4 years	p-values	1st Session T = 0	2nd Session T = 6–13 weeks	p-values
Number (males)	11 (6 males)	11 (6 males)	8(5males)		10 (5 males)	10 (5 males)	
Age (years)	59.2 (±9.7)		63 (±9.4)		60.9 (±6.8)		
Disease duration (years)	3.0				not applicable		
UPDRS motor score (SD)	24 (±9.2)	18 (±7.1)	23 (±5.0)	1:p = 0.006 (n = 11) 2: p = 0.05 (n = 8)	0.1 (±0.3)		
MMSE (SD)	30 (±0.7)		29(±1.2)	p = 0.18	29.6 (±0.8)		
BIS	65.0 (±5.4)		64.5 (±6.8)	p = 0.44	65.3 (±5.8)		
GAQ	0.3 (±1.1)		0.5 (±0.9)	p = 1.00	0.25 (±0.64)		
BDI	5		5.5	p = 0.46			
Mean response times (s)	1.08 (±0.20)	1.01 (±0.14)		p = 0.24	1.02 (±0.20)	1.00 (±0.18)	p = 0.64
Missed card choices % (range)	3.3 (0–11.7)	3.3		p = 0.23	0.8 (0–11.7)	0	p = 0.89

In the patient group, a movement disorder specialist (author C.B), gradually increased the daily treatment dose over several weeks until dopamine agonist therapy induced a stable clinical response with an optimal trade-off between the anti-parkinsonian effects and medication-induced side effects. Five patients started on Pramipexol, five patients started on Ropinirol and one patient started on Rotigotine. Patients had all reached a stable level of dopamine agonists at the time of the follow-up scan. These doses were translated into a total daily levodopa equivalent dose (Tomlinson et al., 2010). The mean levodopa equivalent dose was 183.6 ± 70.9 mg (mean ± SD), showing substantial effective dose variability between patients. From the twelve healthy age-matched controls (5 men, mean age: 60 ± 7 years) we scanned in the first session (Controls_1st,_ henceforth), ten controls (5 men, mean age: 60.9 ± 6.8 years) participated in a second fMRI session after 6–13 weeks (mean duration: 8.6 ± 2.5 weeks, Controls_2nd,_ henceforth), to control for effects related to repeated card game playing. Healthy controls could not be treated with dopamine agonists for ethical reasons. Written consent was obtained from all participants according to the Declaration of Helsinki, and the study was approved by the local research ethics committee. All patients were prospectively recruited from the outpatient clinic for movement disorders at the Department of Neurology, University Medical Centre Hamburg-Eppendorf, Hamburg, Germany.

The diagnosis of PD was made by a movement disorder specialist (CB) in accordance with the UK Parkinson's Disease Society Brain Bank criteria for a clinical diagnosis of PD. The reported onset of motor symptoms ranged from ~0.5 to ~5 years (median symptom duration ~3 years). Prior to scanning, all subjects were examined using the Unified Parkinson's Disease Rating Scale (UPDRS) (Goetz et al., 2007) motor rating scale (part III) and classified according to the Hoehn and Yahr (Hoehn and Yahr, 1967, Goetz et al., 2004) and Schwab and England scales (Schwab and England, 1969). During the first session, global cognitive function was assessed in patients and controls using the mini-mental state examination (MMSE) (Folstein et al., 1975). Symptoms of depression were assessed using Beck Depression Inventory II (BDI, Psychological Corporation, Boston, MA). The Barratt Impulsiveness Scale (BIS) (Patton et al., 1995) and the Gambling addiction questionnaire (GAQ) (Gambling addiction questionnaire of the Berlin Gamblers advisory committee, Düffort, 1986) were used to evaluate risk-taking behaviour or the presence of problematic gambling. Through a clinical interview with the participants, and when possible by asking their spouses, we ascertained that none of the participants had explicit recall of any ICD-like behaviours or reported typical symptoms associated with ICDs such as pathological gambling, compulsive sexual behaviour, compulsive shopping, compulsive/binge eating, or punding (Weintraub et al., 2010), excessive smoking or coffee drinking behaviour. Four years after the study, eight of the original PD patients were available for a clinical interview and behavioural testing. Interview and testing by the clinician comprised recording the development of any ICD associated with medication use, possible change of diagnosis, recent medication and repetition of the following questionnaires BDE, BIS, MMSE and GAQ. The patients were also examined again according to the UPDRS part III.

### 2.2 Gambling task

**Fig. 1:**
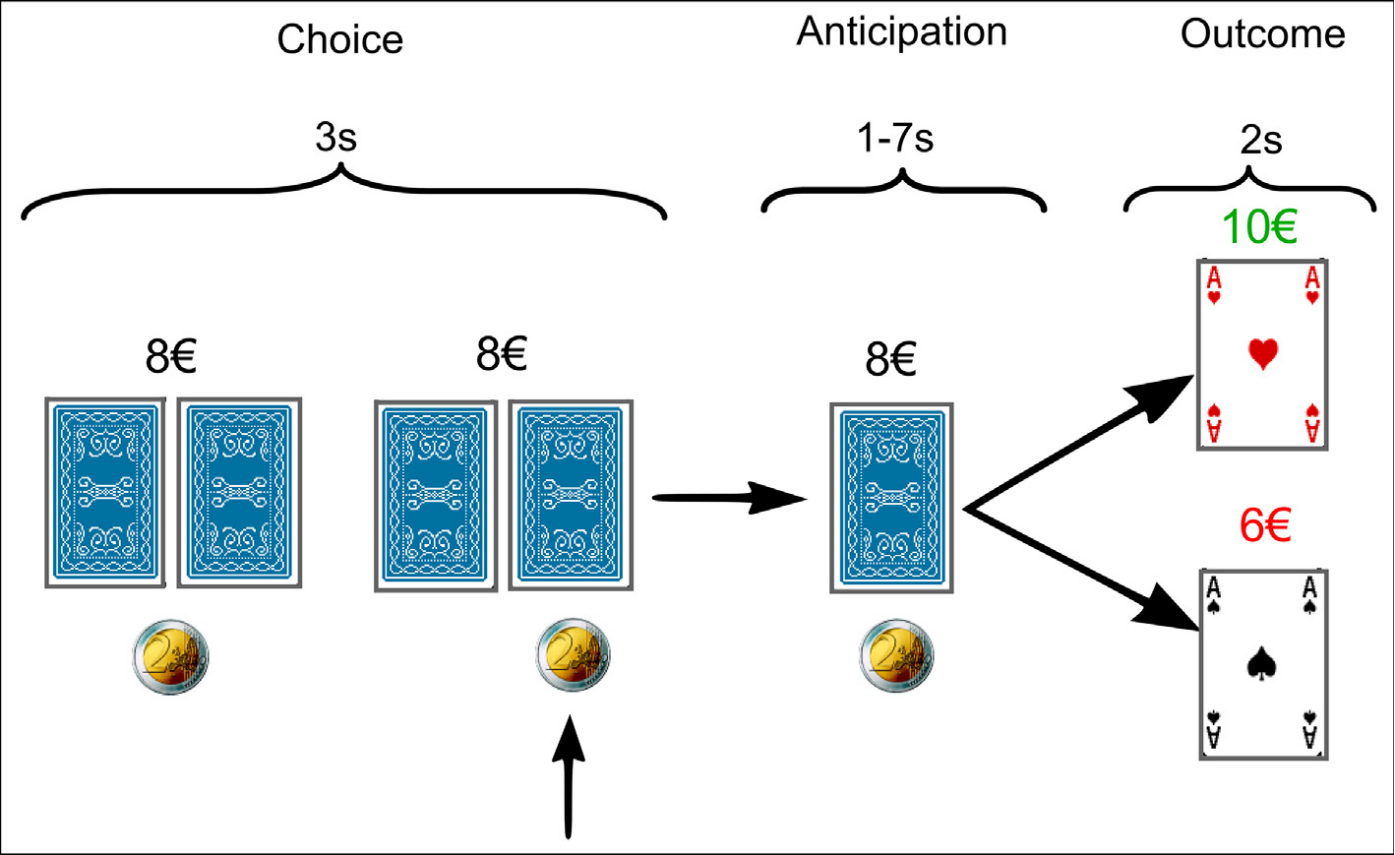
Design of the gambling task. At trial onset (choice phase) participants were dealt two playing cards, alongside iconic representation of the money at stake, either 2 euro (low-stake) or 5 euro (high-stake). Subjects were instructed to select via right-hand button press (index finger for left card, middle for right) which card to select within 3 s. During a jittered period of anticipation (1–7 s) the card was revealed resulting in the monetary gain or loss of the stake (red card signalled winning outcomes, black losses). Between trials there is a jittered period of rest (1–7 s). A cumulative total was displayed above the card at all times during the trial. Probability of winning was 50% and remained constant throughout. (For interpretation of the references to colour in this figure legend, the reader is referred to the web version of this article.)

Participants engaged in a simple two-choice gambling task during fMRI, known to elicit robust reward-related responses in the previously reported regions: the ventral tegmental area, ventral striatum, putamen, caudate nucleus, thalamus, hippocampus and medial orbitofrontal cortex, as identified in (van der Vegt et al., 2013). In each trial, two playing cards were presented alongside iconic representations of the money (Stake_Low_ = 2 euro, or Stake_High_ = 5 euro) that could be lost or won, and participants were required to decide within 3 s, on which card to gamble (Fig. 1). Subjects indicated their decision via a button press (right index for left card, right middle finger for right card) and after a jittered period (1–7 s, uniform distribution) the outcome was revealed. Consecutive trials were separated by identically distributed rest periods. The total cumulative earnings for the whole game (totalling 60 gambles) were constantly displayed above the card during each trial (Mean earnings = 8,80 euro, SD = 1.92). Each subject was randomly assigned to one of five pre-set pseudo-randomised sequences of gambling trials and the probability of winning was independent and stationary at 0.5. The sequences were set up so there would always be a positive monetary reward at the end of the game, and enough variation in the outcomes during the game (cumulative totals also below zero) to reduce the predictability and make it more attractive to play. Stimuli were presented via back-projection viewed via a head-coil mirror. Task presentation and recording of behavioural responses were performed using the software Presentation (Neurobehavioral Systems, Inc., Albany, USA). Before scanning, subjects received a standardized verbal description of the task. When necessary this was adjusted according to the cognitive ability of each patient. Specifically, they were instructed not to press the same button constantly, and truthfully informed of the outcome contingencies (red = win, black = lose), the probabilities, and that the cumulative total would be realised in physical currency at the end of the gambling task. To ensure task competence, subjects were trained on the task outside the scanner (10–15 trials), immediately prior to each fMRI session.

### 2.3 MRI data acquisition and analysis

Whole-brain MR scanning was performed on a 3 Tesla MR Scanner (Siemens Trio, Siemens, Erlangen, Germany) with a 12-channel head coil. Thirty-eight transversal slices (slice-thickness 3 mm) were acquired in each volume (repetition time: 2.5 s; echo time 34 ms; flip angle: 90°: field of view 216 mm) using gradient echo T2*-weighted echo planar imaging. The first three volumes of each fMRI run were discarded to eliminate T1 saturation effects. High-resolution T1-weighted images (1 mm^3^ voxel size) were acquired for each subject, using a magnetization prepared rapid gradient echo sequence. Data pre-processing and analysis were performed using SPM8 software (Wellcome Department of Imaging Neuroscience, London, UK). The pre-processing steps consisted of realignment (rigid body motion correction), segmentation of the high-resolution T1 image, to which the functional images then were co-registered. All images were spatially normalized to Montreal Neurological Institute (MNI) space using the normalization parameters obtained from the unified segmentation procedure and subsequently smoothed with an isotropic Gaussian kernel with 8 mm full-width at half maximum (Ashburner and Friston, 2005).

First-level data analysis was performed for each subject using the general linear model (GLM). Events of interest were modelled as stick functions and convolved with a canonical hemodynamic response function, as implemented in SPM8 (www.fil.ion.ucl.ac.uk/spm/software/spm8/). To model the choice phase, we constructed two regressors each time-locked to the onset of the choice to independently model Stake_High_ and Stake_Low_ trials (van der Vegt et al., 2013). Note that the choice phase included both the button-press and a period of outcome anticipation, which could not be separated in this design due to their collinearity. To model the outcome phase, we constructed four regressors, again time-locked to the onset of the outcome, for all factorial combinations of outcome (win vs. loss), and value (high vs. low). Residual effects of movement after rigid body realignment were modelled by a twenty-four parameter Volterra expansion (Friston et al., 1996) of the movement parameters and treated as nuisance regressors in the GLM. This filter contains the six motion parameters estimated from the rigid body realignment procedure as well as the parameters from the previous volume to account for spin history effects. The filter was expanded to second order by squaring the movement parameters, resulting in 24 additional free parameters that were estimated by the GLM.

After estimation of the first-level model, the planned contrasts for each event type were computed using one sample t-tests (unless otherwise specified). The contrast images from each subject were entered in a group-level random effects analysis. We created a first-order parametric linear t-contrast for [Wins vs. Losses]: high win > low win > low loss > high loss and with this estimated the effect of condition [Wins vs. Losses] at the first level for the groups separately and between sessions [PD: ON versus OFF] [Controls: 2nd versus 1st] after this the interaction between groups [PD versus Controls] could be calculated on the second level with a two sample *t*-test. We also used the flexible multifactorial design on the second level for subject × group × condition analyses. Unless otherwise stated, all statistical inferences reported for neuroimaging data are at a voxel wise significance threshold of p < 0.05 (FWE, with small volume correction over the ROI). We used one ROI consisting of the bilateral ventral striatum, caudate nucleus, thalamus, hippocampus, parahippocampal area and medial orbitofrontal cortex, which was created via the WFU Pickatlas (Maldjian et al., 2003). Since healthy controls did not receive dopamine agonists the present design does not constitute a balanced factorial design, which means that the interaction of diagnostic class and treatment cannot be estimated.

For visualization purposes the regional BOLD signal changes were computed using the rfx plot toolbox (Glascher, 2009) and MNI coordinates of the voxels indicating the regional maxima. For correlation analyses UPDRS motor scores and dopamine-agonists dosage were taken to the random effects level as covariates of interest, with age as a nuisance covariate. Reaction times and other behavioural data were analysed using SPSS (SPSS for Windows Rel. 18.0.0.2009. Chicago: SPSS Inc.). For behavioural data analyses, significance thresholds were set at *p <* 0.05, group data are given as mean ± standard deviation (SD), and all Confidence intervals are reported at the 95% level, unless otherwise stated.

## 3 Results

### 3.1 Behavioural data and motor scores

We ended up studying 11 patients and 10 controls to be able to make a comparison within and between groups. The duration between the first and second scanning session for PD patients and healthy controls was equal (PD 8.4 ± 2.3 weeks, Controls 8.6 ± 2.5 weeks; two-sample T-test: t(19) = 0.22 p = 0.83, 95% CI [−2.4–1.97). The median UPDRS motor score of the 11 PD_OFF_ patients decreased significantly from 24 (UPDRS_OFF_) to 18 (UPDRS_ON_) after several weeks of treatment with dopamine agonists (Wilcoxon Signed-ranks test (WSRT); Z = 2.76, p = 0.006, Table 1). The eight patients who were reassessed after four years had a median UPDRS motor score of 23 (UPDRS_FOLLOW-UP_), which was significantly higher than the UPDRS_ON_ (Z = 1.96, p = 0.050, Table 1). There was no significant correlation between the dose of dopamine agonists and ΔUPDRS_OFF vs ON_ (r (11) = −0.21, p = 0.530). In the PD_OFF_ condition, clinical tests showed no evidence of depressive disorder, dementia, risk-taking or addictive behaviour. At the 4-year follow up, these scores did not differ significantly relative to the initial scores for depression, dementia or risk-taking behaviour. History taking after four years of follow-up showed that none of the eight patients had developed any ICDs associated with medication use. Also, the diagnosis had not changed (Table 1).

Overall response times for the gambling choices did not differ significantly between the two conditions for PD patients (PD_OFF_: 1.08 s ± 0.20 s versus PD_ON_: 1.01 s ± 0.14 s; Paired samples *T*-test: t(10) = 1.25, p = 0.24, 95% CI [−0.06–0.20]) and the 10 controls (Controls_1st_: 1.02 s ± 0.20 s versus Controls_2nd_ 1.00 ± 0.18 s; t(9) = 0.49, p = 0.64, 95% CI [−0.12–0.17). There was also no significant group difference in response times for the PD_OFF_ condition compared to Controls_1st_ (PD_OFF_: 1.06 s ± 0.19 s versus Controls_1st_1.08 s ± 0.23 s; two-sample *T*-test: t(23) = 0.24p = 0.81, 95% CI [−0.16–0.20]) or the PD_ON_ phase compared to Controls_2nd_ (PD_ON_: 1.01 s ± 0.14 s versus Controls_2nd_: 1.00 s ± 0.18 s; t(19) = 0.30, p = 0.77, 95% CI [0.20–0.69]). The percentage of trials in which the choice period expired without a choice was negligible (median, PD_OFF_ = 3.3%, Controls_1st_ = 0.8%, PD_ON_ = 3.3%, Controls_2nd_ = 0%) and was not significantly different OFF versus ON treatment for the PD patients (WSRT (%error-PD_ON_ – %error-PD_OFF_) Z = 1.19, p = 0.23) or the controls (WSRT (%error-Controls_2nd_- %error-Controls_1st_); Z = 0.14, p = 0.89). There was also no significant difference between groups for the PD_OFF_ compared to Controls _1st_ (Mann-Whitney *U* test, Z = 1.44, p = 0.15, Controls_1st_ mean rank: 10.88, PD_OFF_ mean rank: 14.96). However, there was a significant difference in percentage of trials in which the choice period expired without a choice for the PD_ON_ compared to Controls_2nd_ (Mann-Whitney *U* test, Z = 2.22, p = 0.03, Controls_2nd_ mean rank: 8.0, PD_ON_ mean rank: 13.73). See Table 1.

### 3.2 fMRI results

**Fig. 2:**
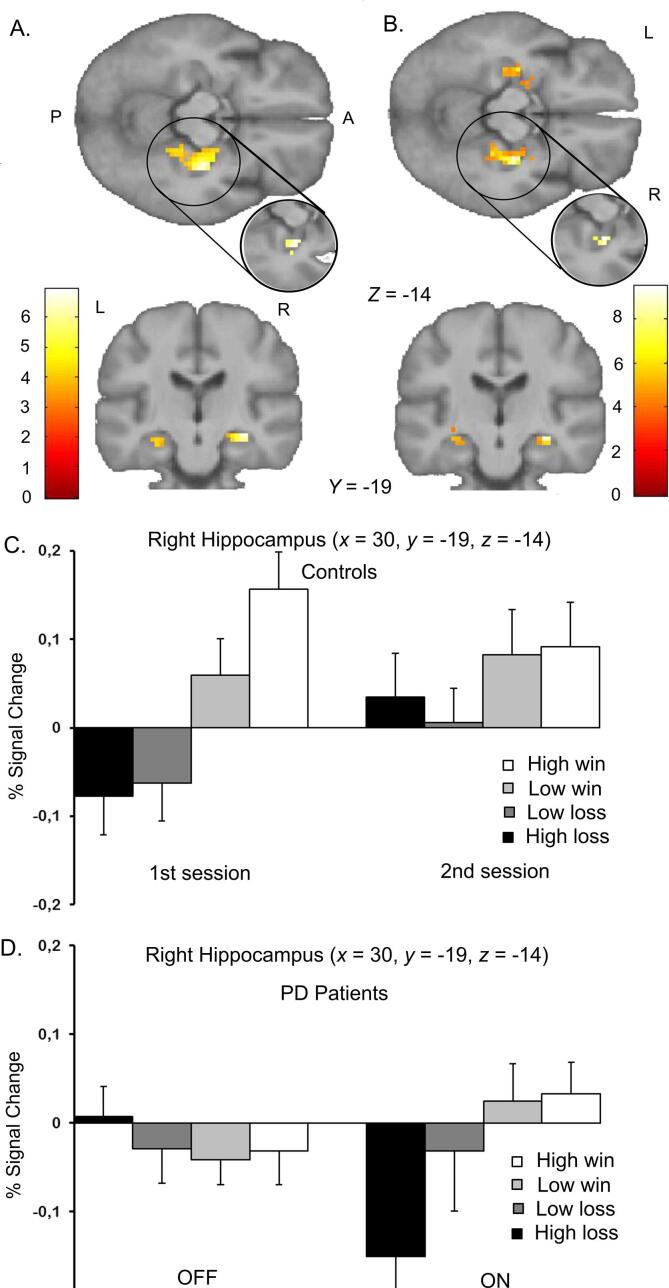
Dopamine-dependent responses to reward outcome in Parkinson’s patients versus controls. A. Statistical parametric maps displaying the right hippocampus showing a stronger increase in activity with reward outcome value for patients than for controls over the two sessions [PD_ON_ > PD_OFF_] versus [Controls_2nd_ > Controls_1st_]. Significant value related differences in Parkinson’s patients on dopamine agonists are present in the right hippocampus. The statistical maps are thresholded at p < 0.001 (unc.), the cut-out circle shows the activity thresholded at p < 0.05 small volume corrected within the ROI. The bar gives the colour coding of T-values for each voxel. (P: posterior, A: anterior, L: left, R: right). B. Statistical parametric maps displaying the hippocampus bilaterally showing a stronger increase in activity with reward outcome value PD_ON_ compared to PD_OFF_. Significant value related differences in Parkinson’s patients on dopamine agonists are present in the right and left hippocampus. The statistical maps are thresholded at p < 0.001 (unc.), the cut-out circle shows the activity thresholded at p < 0.05 FWE-corrected within the ROI. The bar gives the colour coding of T-values for each voxel. (P: posterior, A: anterior, L: left, R: right). C & D. Parameter estimates for outcome-related activity for regional peak activation in the right hippocampus in healthy controls (C) and Parkinson’s patients (D) for the separate sessions. Healthy controls (Controls_1st_) show a clear linear reward value related activity in the right hippocampus in the first session, which is less linear in the second session (Controls_2nd_). In the PD_OFF_ condition there is a flattened (non-linear) neural response with outcome in the right hippocampus, where the clear linear reward value related activity is restored after being on dopamine agonists (PD_ON_).

In PD patients, the effect of dopaminergic treatment [PD_ON_ > PD_OFF_], compared to the controls [Controls_2nd_ > Controls_1st_], was associated with a significantly stronger linear increase of regional activity with reward outcome in the right hippocampus (Fig. 2A, t = 6.93, p = 0.002, FWE corrected, MNI [30,-19,-14]). Anatomically, the cluster of voxels was not restricted to any one hippocampal subfield. The cluster (15 voxels, with a cluster defining threshold of p < 0.001 unc.) had an overlap of 24% with the Cornu Ammonis (CA) 3 region of the right hippocampus, 11% with the right CA2 region, and 11% with the right dentate gyrus. Subfields here are as specified in the Anatomy Toolbox, which is based on cytoarchitectonic mapping of the hippocampus in 10 human post-mortem brains (Amunts et al., 2005). The converse comparisons, in all possible directions, did not show any significant responses in the region of interest.

Within-group analyses [PD_ON_ > PD_OFF_] revealed that dopaminergic treatment increased hippocampal reward responsivity in PD patients. As previously reported (van der Vegt et al., 2013), in the PD_OFF_ condition we detected no regions where activity reflected the magnitude of reward outcome. Here we report that after several weeks of dopaminergic treatment the right hippocampus was the only region where linear reward responsivity was increased. This effect was most visible as a stronger negative BOLD response to high losses. However, all responses to the different stakes were stronger in [PD_ON_ > PD_OFF_] (Fig. 2B, (t = 9.49, p = 0.003, FWE corrected, MNI [30,-19,-14]) resulting in a more positive linear scaling of regional activity with reward outcome. In healthy controls, the scaling of regional activity to reward outcome did not differ significantly between the first and second MRI measurement in the set ROI, or using exploratory whole-brain analyses. Unlike in the first scanning session comparing PD_OFF_ with Controls_1st_ (van der Vegt et al., 2013), there were no significant differences in the linear scaling of brain activity to reward outcome comparing PD_ON_ to Controls_2nd_.

To test whether this stronger linear effect was lateralised to the right, we performed an exploratory test on a region of interest restricted to the left hippocampus. At a lower significance threshold (p < 0.001 unc.) we found differential reward-related effects also in the left hippocampus. Although we are careful to interpret non-significant trends, this suggests that the reward-related effects we report above are not lateralised to the right hippocampus only (Fig. 2).

**Fig. 3:**
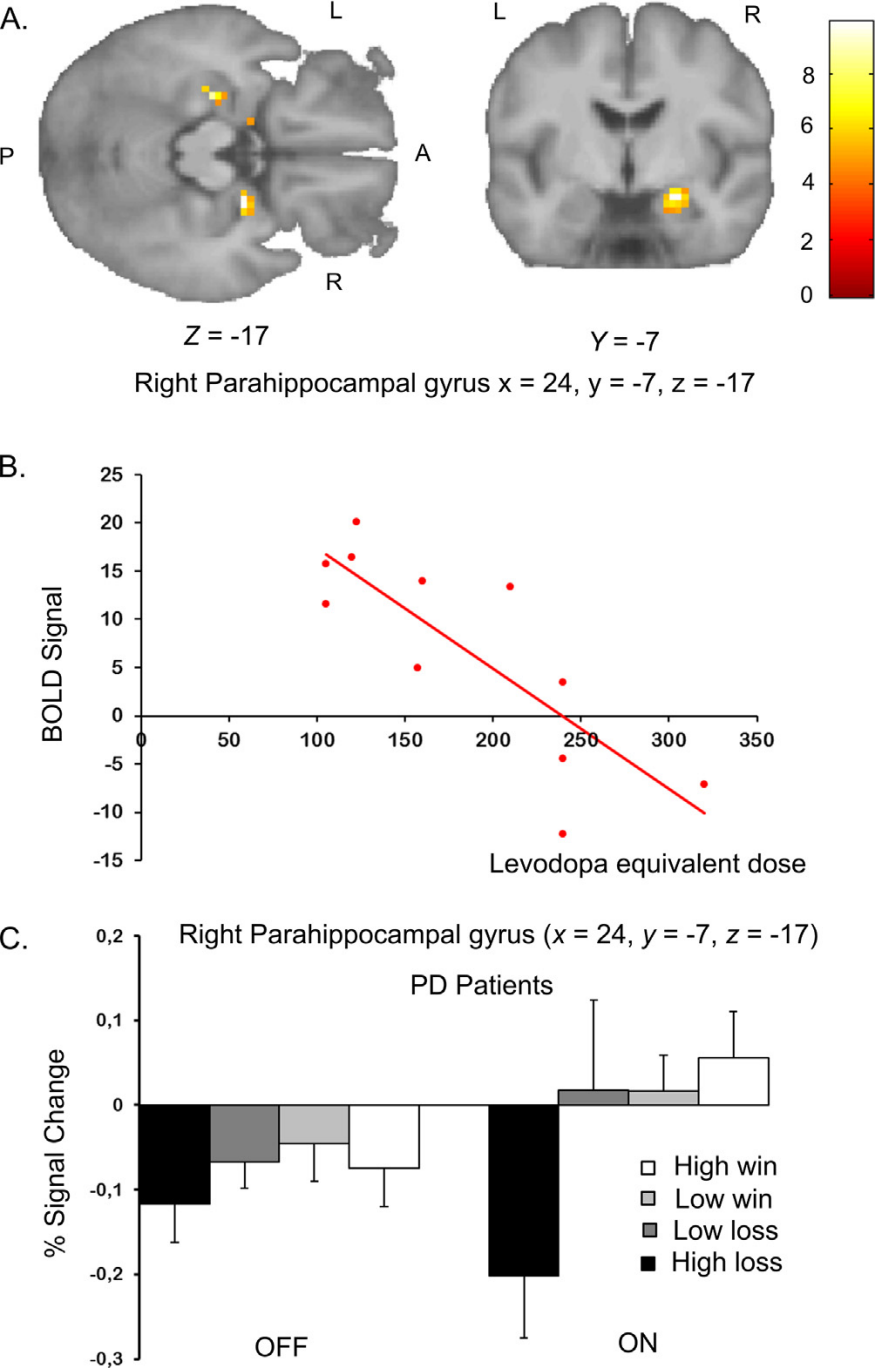
Relationship between reward-value related activity in the right parahippocampal gyrus and drug dose. A. Statistical parametric maps showing a cluster in the right parahippocampal gyrus where reward related activity showed a negative linear relationship with the individual dopamine-agonists dosage independent of the UPDRS score and age [PD_ON_ > PD_OFF_]. The statistical maps are thresholded at p < 0.001 (unc.). The bar reflects the colour coding of T-values for each voxel. (L: left, R: right). B. The scatter plot illustrates the negative linear decrease in the estimated reward related BOLD response with the motor individual dopamine-agonists dosage for the peak voxel of the right parahippocampal gyrus in Parkinson’s patients. It indicates that patients on a lower dose of dopamine-agonists showed a more linear reward value related activity which resembles the response profile of healthy controls. C. Parameter estimates for outcome-related activity for regional peak activation in the right parahippocampal gyrus in Parkinson’s patients for the separate sessions. In the PD_OFF_ condition there is a flattened (non-linear) neural response with outcome in the right parahippocampal gyrus, where the clear linear reward value related activity is restored after being on dopamine agonists (PD_ON_).

In the PD group [PD_ON_ > PD_OFF_], the increased reward responsivity of the right parahippocampal region correlated negatively with the individual levodopa equivalent dose (Fig. 3, t = 9.84, p = 0.011, FWE corrected, MNI coordinates = [24, −7, −17]) corrected for age and UPDRS. Anatomically the cluster (33 voxels, with a cluster defining threshold of p < 0.001 unc.) has a 48% overlap with the right amygdala. There is a small spatial overlap with the aforementioned activity found in the right hippocampus, this region is situated directly on the anterior edge of the hippocampal region. In other words, the lower the individual levodopa equivalent dose, the greater the sensitivity of the right para-hippocampal gyrus to reward outcome. Neither age nor individual motor improvement, operationalised as a decrease in UPDRS, were correlated with regional changes in reward responsivity in the set ROI (in confirmatory analyses) or other regions (in exploratory whole-brain analyses).

## 4 Discussion

This study builds on previous data where we showed attenuated reward responsivity in a reward-related network in newly diagnosed, drug-naive PD patients [PD_OFF_ > Controls_1st_] (van der Vegt et al., 2013). In the current study, we tested two hypotheses: (1) that dopaminergic treatment would restore the attenuated, mesolimbic and mesocortical response to reward; and (2) that these effects would predict motor performance and correlate with development of neuropsychiatric symptoms associated with dopaminergic overstimulation (i.e. impulse control disorders). Our findings only partially support the first hypothesis, insofar as they show that dopamine agonist therapy re-introduced a positive linear scaling of regional activity with reward outcome in the right hippocampus (Fig. 2A&B). We found no such increase in linear relationship with reward magnitude elsewhere in the mesolimbic or mesocortical systems at a significant level. However, our data do not provide evidence for or against the second hypothesis, given that none of the patients tested developed impulse control disorders in the follow-up period of four years (in 8 out of 11 that were followed for this period of time).

Previous evidence supports the idea that dopaminergic dysfunction of the hippocampus may have a role in the pathophysiology of PD. The hippocampus has been implicated in non-motor symptoms like cognitive problems and impulse control disorders in PD. Emerging data suggest interactions between the dopaminergic systems and the hippocampus in synaptic plasticity, adaptive memory, and motivated behaviour (Calabresi et al. 2013). Specifically, the hippocampal formation receives significant dopaminergic projections, as assessed using post-mortem DAT labelling in primates (Lewis et al., 2001) and 18-F-DOPA labelling in healthy humans (Nagano et al., 2000). PD patients have been observed to have reduced dopamine concentrations in the hippocampus, as assessed using post-mortem analyses (Scatton et al. 1982). Furthermore, the hippocampus is implicated in reward-contingent memory formation in healthy subjects (Mattfeld and Stark, 2015) and is densely connected with the ventral striatum (Helmich et al., 2010, Mattfeld and Stark, 2015). Taken together, the observed effects of dopamine on hippocampal activity may serve to modulate the encoding of episodes into memory (Brzosko et al., 2015, Brzosko et al., 2017, Zannone et al., 2018).

The differential effect of dopaminergic medication on reward processing was strongest in response to high losses, rather than to wins (Fig. 2D). In our previous paper (van der Vegt et al., 2013), we hypothesised that this loss sensitivity would diminish, and gain sensitivity would increase following the initiation of dopamine medication (Frank et al., 2004, Frank et al., 2007, Schott et al., 2007, Bodi et al., 2009). Contrary to our predictions, dopaminergic treatment mainly increased loss sensitivity activity in right hippocampus and right parahippocampus. For the parahippocampal cluster, the change scaled with individual dose (see below). A previous fMRI study in healthy individuals showed that the motivational context may influence the sensitivity of mesial temporal area to surprising outcomes (Murty et al., 2016). Reward motivation (monetary reward vs. no reward) enhanced sensitivity in a (left) hippocampal cluster to surprise, while punishment motivation (electrical shock vs no shock) enhanced the sensitivity of a (right) parahippocampal cluster. It is possible that “punishment motivation” (avoiding monetary loss) was preponderant in medicated PD patients, resulting in a more prominent treatment effect in trials with large loss outcomes.

We further hypothesised that restoration of reward responsivity by dopaminergic treatment would be predictive of motor performance and the emergence of impulse control symptoms. Motor performance improved initially after the start of dopaminergic treatment; however, after 4 years of disease progression, patients deteriorated again under treatment with dopaminergic drugs. None of the 8 (out of 11) patients, where follow up was possible, developed ICD’s after 4 years on dopamine-replacement therapy. No data were available on the other three. This is somewhat surprising, given previous estimates that the incidence rate is up to 35% (2,6–34,8%) after 6 months (Garcia-Ruiz et al., 2014, Voon et al., 2017, Grall-Bronnec et al., 2018). Individual susceptibility factors, other than the ones taken into account here, could have played a role in this, however the small sample size makes it impossible to adequately test for this.

An unexpected exploratory finding was the negative correlation of reward-related activity in the right parahippocampal gyrus with the dopamine-agonists dosage (Fig. 3). It indicates that patients on a lower dose of dopamine-agonists showed reward-related responses that resembled more closely healthy subjects. Whilst it may be natural to assume that a lower dose was indicative of lower symptom severity, we found that this effect was independent of the preceding UPDRS score. This negative correlation could fit with the inverted U-shaped relationship between dopamine levels and mesolimbic brain activity, as predicted by the dopamine-overdose hypothesis (Cools, 2006). Possibly, this would suggest that the patients we studied were on the descending slope of this curve, which fits with the fact that we studied very early, drug-naïve patients.

**Fig. 4:**
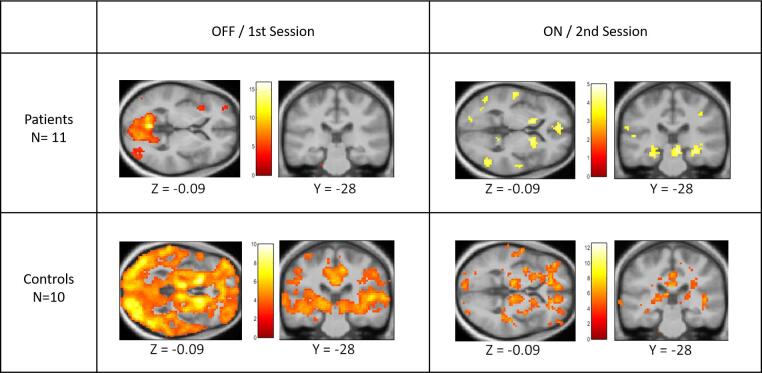
Full pattern of reward-value related activity in patients and controls for the separate sessions. Statistical parametric maps displaying the full pattern of reward-value related activity, that is the linear effect of outcome value [high losses to high wins], separately for each of the two groups (PD, Controls) and for each of the two sessions (PDOFF, PDON, Controls1st, Controls2nd). The statistical maps are thresholded at p < 0.001 (unc.). The bar gives the colour coding of T-values for each voxel.

The main limitation of this study derives from its small sample size (n = 11) that resulted from the difficulty in obtaining drug-naïve patients both able and willing to participate in a longitudinal imaging study Fig. 4. The results should thus be interpreted in the context of several caveats understood to impact on studies that are underpowered for detecting clinically relevant effect sizes. Precautions in interpretation should be balanced against the relative rarity of the observations, and the potential clinical importance of such observations, as it may be the case here with a rare example of drug-naïve PD patients. Furthermore, due to ethical restrictions, the current study was neither placebo-controlled nor double blinded, and therefore, strictly speaking, drug dependent reward-signalling effects cannot be separated from a putative placebo effect. However, unlike previously studied PD patients, our drug-naïve PD patients had never experienced the effect of dopaminergic medication before they were examined in the OFF session, to reduce the influence of the placebo effect (de la Fuente-Fernandez et al., 2001, de la Fuente-Fernandez and Stoessl, 2002). More specifically, the patients did not have negative expectations for an eventual post-medication OFF state (given that they were drug-naïve), and they did not have exaggerated expectations for the ON state (given that they received their own medication). This is an advantage of our design over other studies where dopaminergic medication was halted. The inclusion of drug-naive patients also avoided confounding long-term effects of previous dopaminergic therapy. Finally, our design was optimized to detect linear cerebral responses as a function of reward, and therefore we were not able to detect potential non-linearities in the reward responses (Voon et al., 2010). For future research, it would be interesting to look at the effect of dopamine agonists in a more extensive and structured way. Administering the same dopamine agonist in equal steps in a larger group of drug naïve patients for a longer period of time would make it possible to better correlate the effect of medication with the reward related activity and probably have a more reliable outlook on the development of ICDs over time.

## 5 Conclusions

We found that in drug naïve Parkinson’s disease patients, the attenuated reward responsivity observed on diagnosis was only increased in a subset of reward-responsive regions after several weeks of treatment with dopamine agonist therapy. These drug-dependent reward effects were largest in the hippocampal and parahippocampal cortex, and most pronounced in patients treated with low doses of dopamine agonists. These findings could be considered partially consistent with the dopamine overdose hypothesis, though more stringent tests of the theory would necessitate high-powered double-blinded replication in larger samples, provided ethical problems can be circumvented. Specifically, whether reward-assays (such as this protocol) can be used to predict the emergence of impulse control disorders will be the focus for future work.

## Funding

This work was supported by the 10.13039/501100003246Netherlands Organisation for Scientific Research (NWO; VIDI grant No. 16.076.352 to B.R.B.) and a grant of excellence by the 10.13039/501100003554Lundbeck Foundation on the Control of Action (ContAct; grant No. R59 A5399 to H.R.S.). H.R.S. holds a 5-year professorship in precision medicine at the Faculty of Health Sciences and Medicine, University of Copenhagen which is sponsored by the 10.13039/501100003554Lundbeck Foundation (Grant Nr. R186-2015–2138).

## CRediT authorship contribution statement

**Joyce P.M. Vegt:** Conceptualization, Resources, Investigation, Formal analysis, Visualization, Writing - original draft. **Oliver J. Hulme:** Formal analysis, Writing - original draft, Validation, Writing - review & editing. **Kristoffer H. Madsen:** Formal analysis, Software, Writing - review & editing. **Carsten Buhmann:** Resources, Writing - review & editing. **Bastiaan R. Bloem:** Conceptualization, Writing - review & editing, Funding acquisition, Supervision. **Alexander Münchau:** Conceptualization, Writing - review & editing. **Rick C. Helmich:** Writing - review & editing, Supervision, Validation. **Hartwig R. Siebner:** Conceptualization, Methodology, Validation, Writing - review & editing, Funding acquisition, Supervision.

## Declaration of Competing Interest

The authors declare the following financial interests/personal relationships which may be considered as potential competing interests: Hartwig R. Siebner has received honoraria as speaker from Sanofi Genzyme, Denmark and Novartis, Denmark, as consultant from Sanofi Genzyme, Denmark and as senior editor (NeuroImage) and editor-in-chief (Neuroimage Clinical) from Elsevier Publishers, Amsterdam, The Netherlands. He has received royalties as book editor from Springer Publishers, Stuttgart, Germany. Rick Helmich serves on the clinical advisory board of Cadent Therapeutics and received honoraria from AbbVie. Bas Bloem has received funding from the Parkinson’s Foundation, the Netherlands Organization for Scientific Research, Stichting Internationaal Parkinson Fonds and the Michael J Fox Foundation. The Center of Expertise for Parkinson & Movement Disorders of the Radboud University Medical Center was supported by a center of excellence grant of the Parkinson's Foundation. Prof. Alexander Münchau has received commercial research support from Pharm Allergan, Ipsen, Merz Pharmaceuticals, Actelion and honoraria for lectures from Pharm Allergan, Ipsen, Merz Pharmaceuticals, Actelion, GlaxoSmithKline, Desitin and Teva. He receives royalties for the book Neurogenetics (Oxford University Press).
