# Supplementary material for: Dopamine agonist treatment increases sensitivity to gamble outcomes in the hippocampus in de novo Parkinson’s disease
Source: Neuroimage Clin. 2020 Jul 25;28:102362. doi: 10.1016/j.nicl.2020.102362 (PMC7453137; doi:10.1016/j.nicl.2020.102362)
Supplement: Supplementary data 1 [file mmc1.docx]

Supplementary materials

Figure 4

**Reward related responses**

The linear effect of outcome value of the separate regressors set for each outcome value (from high losses to high wins). Statistical parametric maps separately for each of the two groups (PD patients, controls), and for each of the two sessions (Patients: OFF/ON, controls: 1^st^/2^nd^). The statistical maps are thresholded at p < 0.001 (unc.). The bar gives the colour coding of T-values for each voxel.
